# Factors Associated with Delays in Treatment Initiation after Tuberculosis Diagnosis in Two Districts of India

**DOI:** 10.1371/journal.pone.0039040

**Published:** 2012-07-09

**Authors:** Durba Paul, Arundhathi Busireddy, Sharath Burugina Nagaraja, Srinath Satyanarayana, Puneet Kumar Dewan, Sreenivas Achutan Nair, Silajit Sarkar, Quazi Toufique Ahmed, Shakuntala Sarkar, Sreenivas Rao Motta Shamrao, Anthony David Harries, John Ethan Oeltmann

**Affiliations:** 1 Office of the WHO-Representative in India, World Health Organization (WHO), New Delhi, India; 2 District TB Office, Nalgonda, Andhra Pradesh, India; 3 International Union Against Tuberculosis and Lung Disease, South-East Asia Office, New Delhi, India; 4 District TB Center, Bardhaman, West Bengal, India; 5 Directorate of Health Services, State TB Office, Government of Andhra Pradesh, India; 6 International Union Against Tuberculosis and Lung Disease, Paris, France; 7 London School of Hygiene and Tropical Medicine, London, United Kingdom; 8 Division of TB Elimination, United States Centers for Disease Control and Prevention, Atlanta, Georgia, United States of America; McGill University, Canada

## Abstract

**Background:**

Excessive time between diagnosis and initiation of tuberculosis (TB) treatment contributes to ongoing TB transmission and should be minimized. In India, Revised National TB Control Programme (RNTCP) focuses on indicator start of treatment within 7 days of diagnosis for patients with sputum smear-positive PTB for monitoring DOTS implementation.

**Objectives:**

To determine length of time between diagnosis and initiation of treatment and factors associated with delays of more than 7 days in smear-positive pulmonary TB.

**Methods:**

Using existing programme records such as the TB Register, treatment cards, and the laboratory register, we conducted a retrospective cohort study of all patients with smear-positive pulmonary TB registered from July-September 2010 in two districts in India. A random sample of patients with pulmonary TB who experienced treatment delay of more than 7 days was interviewed using structured questionnaire.

**Results:**

2027 of 3411 patients registered with pulmonary TB were smear-positive. 711(35%) patients had >7 days between diagnosis and treatment and 262(13%) had delays >15 days. Mean duration between TB diagnosis and treatment initiation was 8 days (range = 0–128 days). Odds of treatment delay >7 days was 1.8 times more likely among those who had been previously treated (95% confidence interval [CI] 1.5–2.3) and 1.6 (95% CI 1.3–1.8) times more likely among those diagnosed in health facilities without microscopy centers. The main factors associated with a delay >7 days were: patient reluctance to start a re-treatment regimen, patients seeking second opinions, delay in transportation of drugs to the DOT centers and delay in initial home visits. To conclude, treatment delay >7 days was associated with a number of factors that included history of previous treatment and absence of TB diagnostic services in the local health facility. Decentralized diagnostic facilities and improved referral procedures may reduce such treatment delays.

## Introduction

India has the highest burden of Tuberculosis (TB) in the world, accounting for approximately one fifth of the global incidence. An estimated 2.3 million cases occur annually, of which 0.8 million are sputum-smear positive [1]. Someone with untreated smear positive pulmonary TB (PTB) can continue to infect others until effective treatment is initiated. The best strategy to break the chain of TB transmission is early diagnosis, initiation, and completion of treatment [2], [3].

Delays in patients reporting to health facilities with symptoms and in health care workers reaching a diagnosis and initiation of TB treatment all lead to continued transmission of infection. Studies have documented the length of time from onset of symptoms to the diagnosis of PTB. However, few studies have described the extent to which the health system contributes to delays that may occur from the time of PTB diagnosis to the start of anti-tuberculosis treatment, and few have reported on risk factors for these delays [4], [5].

One of the indicators of the quality of DOTS implementation under the Revised National TB Control Programme (RNTCP) of India is start of treatment within 7 days of diagnosis for patients with sputum smear-positive PTB [6]. Nationwide in 2010, 86% of patients registered with smear-positive PTB were put on anti-tuberculosis treatment within 7 days of diagnosis. This figure varies throughout the country, and in some districts only 60% of patients were initiated on treatment within 7 days [7].

To better understand factors associated with delays between diagnosis and treatment, we conducted a study in two districts of India to assess the length of time between diagnosis and treatment of smear-positive PTB. We also sought to identify risk factors associated with delays of more than 7 days from the patient’s perspective.

## Methods

### Study Design

This was a retrospective survey involving a record review of TB registers, laboratory registers and treatment cards of all patients with smear-positive pulmonary TB registered from July-September 2010 in two districts in India. This was followed by structured cross-sectional interviews of a random sample of patients with PTB. Patients were using simple random sampling from a complete list of patients who experienced a delay of more than 7 days between diagnosis and start of treatment.

### Setting

We conducted the study in two rural districts of two states in India: Bardhaman, West Bengal (population 7.8 million) and Nalgonda, Andhra Pradesh (population 3.5 million).

In India there are different types of health care institutions where pulmonary TB is diagnosed using sputum smear examination. The institutions vary from tertiary level hospitals such as Medical colleges, District TB centers at district level, Tuberculosis Units at sub district level, and Peripheral Health Institutions (PHIs). There are two types of PHIs, with and without sputum smear microscopy services. Microscopy services are placed in the busier government health facilities, and cover approximately 100,000 population. All persons suspected of having PTB are referred to a Designated Microscopy Centre (DMC) for sputum smear examination where two sputum samples (one spot and one early morning) are collected and examined for acid-fast bacilli (AFB) using light binocular microscopy. People suspected of having PTB seen in a PHI without microscopy must travel to a microscopy center for sputum smear examination and bring those results back to the local area PHI for the diagnosis and categorization. People residing in the area of a PHI with microscopy have sputum smear examination, categorization, and diagnosis carried out at the same location.

**Figure 1:**
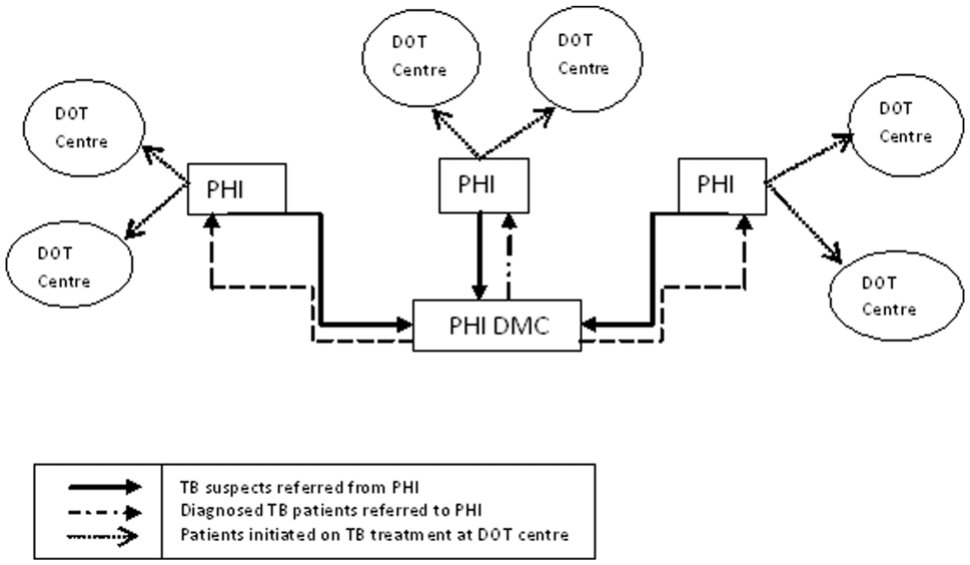
Diagram showing the initiation of TB treatment at DOT centers of Peripheral Health Institutions (PHIs).

Providers at PHI’s prescribe but do not initiate anti-TB treatment. All patients report to their local area DOT Centers to start treatment as shown in Fig. 1. DOT centers are highly decentralized. They are not stand–alone facilities, but rather serve as any treatment delivery point from where a DOT provider operates (e.g. home of a village health worker, community volunteer, local sub-centre where a nurse operates, etc). The Health worker covering the area of the DOT Center collects the anti-TB medication from the PHI, conducts an Initial Home visit (IHV) for verifying the address, and counsels the patient. S/he then identifies a DOT Provider for the patient and starts the patient on treatment. The steps involved from diagnosis to appropriate treatment for both those initially seen at a PHI with microscopy and those seen in a PHI without microscopy are the same except for the fact that those seen in a PHI without sputum microscopy must travel to get their sputum examined at a DMC [8].

### Study Participants

This study included all patients with sputum smear positive PTB registered from 1^st^ July to 30^th^ September 2010 in the participating two districts. Any patient with at least one sputum smear found to be positive for acid-fast bacilli was defined as having smear positive PTB.

### Variables and Data Collection, Data Analysis and Statistics

The number of days between the date of diagnosis of PTB and the date of initiation of treatment was collected from the laboratory register, the TB register and treatment cards. The outcome variable was delayed treatment initiation, defined as treatment starting more than 7 days after TB diagnosis. Exposure variables included: age, sex, health system level of microscopy facility [District TB Centre (DTC-DMC), TB Unit (TU-DMC), DMC of other districts, Medical College DMC, other DMC], type of TB [(new smear positive (NSP), retreatment TB (RT)], and presence of microscopy at the health facility where the patient was diagnosed (PHI with or without microscopy). We used crude and adjusted odds ratios and 95% confidence limits to assess the relation between these categorical variables and delayed initiation of treatment. For univariate analysis, we calculated maximum likelihood estimates of matched odds ratios and their exact 95% confidence intervals. We calculated P-values using the Mantel-Haenszel method or Fisher’s exact test. We determined a P-value of <0.05 was statistically significant and all tests of significance were two tailed. Independent variables which were significantly associated with our outcome of interest during the crude analyses were all included in one logistic regression model to calculate adjusted associations.

### Structured Interviews of a Sample of Patients with Delayed Treatment Initiation

Using simple random sampling we selected 75 patients from each district (150 in total) who had delayed treatment initiation >7 days. We interviewed patients using a structured questionnaire to gather additional descriptive information regarding delayed initiation of treatment that was unavailable from routine records. Participants were interviewed at their home and were still receiving medication for TB. Interviews were conducted in October, November, and December of 2010. Repeated visits were made if the patients were not available during the first house visits, all patients were interviewed during this period. Exposure variables collected were duration between first and second sample collection, duration in days between second sputum collection and collection of smear examination reports, duration between report collection and initial home visit by health worker, and duration between initial home visit and initiation of treatment.

### Ethical Considerations

We received informed consent from all patients that participated in the interviews. Ethical approval was obtained from the Institutional Ethics Committee of National Tuberculosis Institute (NTI), Bangalore and the Ethics Advisory Group of the International Union against Tuberculosis and Lung Disease.

## Results

There were 2027 patients with PTB (79% male), whose median age was 39 years. The distribution of time from diagnosis to initiation of treatment is shown in Table 1. Overall, 711 (35%) patients were initiated on treatment after 7 days, and this included 262 (13%) with treatment delay of >15 days.

**Table 1:** Time from diagnosis to initiation of TB treatment in patients with smear positive TB under RNTCP, during July–Sept 2010, in Bardhaman (West Bengal) and Nalgonda (Andhra Pradesh) districts.

Number of days	Number of patients (%)
0 to 7	1316 (65)
8 to 14	449(22)
15 to 21	123(6)
Above 21	139(7)
Total	2027(100)

Factors associated with delays in initiation of treatment are shown in Table 2. On univariate and multivariate analyses, the main factors associated with delay were residing in a non-DMC PHI area, undergoing diagnostic microscopy outside their TB Unit/District, and previously being treated for TB.

**Table 2:** Factors associated with delayed initiation of treatment of more than 7 days among patients with smear positive TB under RNTCP, during July–Sept 2010, in Bardhaman (West Bengal) and Nalgonda (Andhra Pradesh) districts.

	Total	Delayed initiation of treatment >7days	Odds ratio (95% CI)	Adjusted odds ratio(95% CI)
Age group (in years)		n (%)		
0–14	22	7 (32)	0.6 (0.2–1.7)	–
15–34	732	251 (34)	0.7 (0.5–1)	–
35–54	852	301 (35)	0.8 (0.5–1.1)	–
55–65	299	101 (34)	0.7 (0.5–1.1)	–
≥65	122	51 (42)	Reference	
Sex				
Male	1597	574 (36)	1.2 (1.0–1.5)	1.1 (0.9–1.4)
Female	430	137 (32)	Reference	
History of prior treatment >1 mo				
Yes (Re-treatment case)	507	229 (45)	1.8 (1.4–2.2)	1.8 (1.5–2.3)
No (New case)	1520	482 (32)	Reference	
HIV-Status				
Positive	50	16 (32)	1.0 (0.5–1.8)	0.9 (0.5–1.8)
Unknown	863	339 (39)	1.4 (1.1–1.7)	1.6 (1.3–1.9)
Negative	1114	356 (32)	Reference	
Type of Diagnostic Facility				
Medical College	195	68 (35)	1.2 (0.8–1.7)	1.6 (1.1–2.4)
Outside district-DMC*	85	39 (46)	1.7 (1.1–2.6)	1.7 (1.1–2.7)
Outside TU-DMC	23	18 (78)	7.1 (2.6–19.3)	7.8 (2.8–21.6)
TU-DMC	824	280 (34)	1.0 (0.8–1.2)	1.1 (0.9–1.3)
Other PHI DMC	900	306 (34)	Reference	
Microscopy in treating unit				
Without microscopy	783	316 (40)	1.5 (1.2–1.8)	1.6 (1.3–1.9)
With microscopy	1244	395 (32)	Reference	

* TU – tuberculosis unit, DMC – Designated Microscopy Centre, PHI – peripheral health institution.

Demographic and clinical factors among the randomly selected participants did not differ significantly from those of the entire study population. The patient and provider related reasons for delays in initiation of treatment are reported from patient interviews with delay in these districts are shown in Table 3. Patients who had treatment delay >7 days reported that delays were mainly due to lack of smear microscopy in the original clinic, reluctance to be put on injectable drugs, seeking a second opinion after the diagnosis and delays in obtaining the reports. Again patients also attributed treatment delay to transportation of drugs from the Peripheral Health Institution to the DOT provider and the compulsory initial home visit by the health worker.

**Table 3:** Major reasons for delay in initiation of treatment as reported from 150 patients who had a delay in the diagnosis of smear positive TB between July–Sept 2010, in Bardhaman (West Bengal) and Nalgonda (Andhra Pradesh) districts.*

Patient related reason:
1. Lack of smear microscopy in the original clinic, with need of referral to a microscopy centre for testing, and loss of time after results during referral back to the original clinic. (130/150)#
2. Seeking a second opinion after the diagnosis has been established. (48/150)#
3. Reluctance to be put on Re-treatment regimen (which contains injectable drugs). (30/150)#
4. Time taken from the first sputum collection at the Designated Microscopy Centre and the collection of reports. (28/150)#
**Provider related reasons (Patient’s perspectives of provider related factors):**
1. Delay in transport of drugs from the Peripheral Health Institution to the DOT centers where the patient is supposed to start his DOT.
2. Delay in programme-mandated initial Home visits by the health workers before the start of treatment.

* Reasons are listed based on the frequency with which they were reported by patients (i.e., the first reason listed was most frequently cited, followed by the second, the third, etc.).

# Multiple answers given by the patients.

## Discussion

This is one of the first studies conducted under programmatic settings in India to address delayed initiation of TB treatment after the time of diagnosis. Four our data from two districts in India, we learned that more than one-third of patients with smear-positive TB had treatment delayed more than 7 days after diagnosis, including 13% with delayed-treatment more than 15 days. Not surprisingly we documented that treatment was more likely to be delayed when diagnosis and treatment were separated. Where the patient lived near the microscopy center performing TB diagnosis, they were quickly initiated on treatment. Where the patient was diagnosed at a center without microscopy or had to be referred for treatment to a local DOT center, delay was more likely to occur. Programme procedural barriers like sharing of timely results with the referring doctor or transportation of the drugs to the DOT center, may exacerbate this geographical association. However, patients who experienced treatment delays frequently reported seeking second opinions especially from private sector which have presence in the local areas, suggesting that treatment delay was driven by more than geography or procedural barriers. Limited confidence in their TB diagnosis may also have played a role.

In few studies conducted elsewhere the core problem in delay of diagnosis and treatment seemed to be a vicious cycle of repeated visits at the same healthcare level, resulting in nonspecific antibiotic treatment and failure to access specialized TB services [4].

There are a number of important implications of this study for tuberculosis control in India. First, patients residing in areas where they do not have diagnostic facilities have a greater chance of delay in the initiation of their treatment, and this is almost certainly due to problems of access that involve distance and time. While microscopy services are already decentralized to the 100,000 person level in India, further decentralizing of laboratory diagnosis in districts may help to reduce delay. This would have to be balanced by considerations in maintaining a sufficient laboratory workload to maintain laboratory technician competence.

Second, patients who are diagnosed outside their own area have an increased likelihood of delay. The RNTCP is meant to adhere to a mechanism of referral and feedback when a patient is diagnosed outside his/her area. This mechanism, based on paper exchange, is time-consuming. Why patients sometimes seek care outside their local area, despite service availability, was not addressed in this study. In this study, the duration between TB diagnosis and treatment initiation was 0–128 days compared to the total delay in diagnosis of pulmonary tuberculosis of 25–185 days in other studies [5].

Third, the transportation of drugs in the patient-wise box for a diagnosed patient to a local DOT center after diagnosis may contribute to delay; this could be addressed through decentralization of treatment stocks, using general health system staff for same-day transportation of the treatment box, or issuing some initial treatment doses from the point of diagnosis to minimize any treatment delay. The Provider related reasons [Table 3] may be due to the vacancy of the staff in that particular area or the staff may be pre-occupied with other health related activities in the PHI.

Fourth, retreatment patients are more likely to experience delays than new patients. There may be a number of contributing factors to this observation, including lack of confidence in diagnosis, seeking second opinions, side effects experienced with previous drug intake, or fear of injectable streptomycin. Such cases should be treated with more concern knowing that they may have had negative previous treatment experiences. In addition, patients previously-treated for TB experienced more treatment delay than new patients, which some patients attributed to concern about having to receive an injectable drug. Though the potential of Private sector laboratories cannot be undermined in the study areas none of the laboratories have been recognized as Designated Microscopy centres by RNTCP. It is undoubted that involving labs in the Public Private Partnership mode could decrease the gap in accessibility to TB diagnosis in the districts.

Finally, a recent study on health seeking behavior of persons with symptoms typical of pulmonary TB [9] showed that patients may not seek health care services at certain centers which are not patient friendly. Ensuring that health care facilities are more approachable and accessible could help patients make more use of these facilities.

### Limitations

This was an operational study and therefore subject to the usual limitations of such research. We could only assess variables which are routinely collected in the registers, and there may be other factors related to delay which we were not able to assess. Furthermore we could not differentiate between patient related delay and provider related delays from the register reviews. We were reassured, however, by the concordance between the findings from record analysis and patient interviews. Finally, as there was no comparison group for the patients who completed the structured interview, we cannot confirm which patient-reported factors were related to delayed treatment initiation. Rather, results from these interviews merely suggest factors related to delayed treatment initiation which deserves further study. Recall bias could exist based on the duration between treatment initiation and time of interview. Health worker interviews would have provided insights into provider related delays. To conclude, treatment delay >7 days in Nalgonda and Bardhaman district were associated with the logistic and geographical separations of diagnosis and treatment. To reduce treatment delay, diagnostic facilities should be made more accessible to the patients. The existing system of referral and feedback for patients diagnosed outside their areas needs to be strengthened. More intensified pretreatment counseling on the necessity of early initiation of treatment is required. Initial home visits need to be undertaken and drugs need to be transported to the DOT centers as soon as the patient is diagnosed at the PHI. Reassuring a patient and providing supportive treatment to all patients, especially retreatment cases, must take account of the practice of seeking second opinions and the hindrances experienced due to side effects experienced during previous drug administration.
